# Exploring the Efficiency of Magnetic Separation and Gravity Concentration for Valorizing Pb-Zn Smelter Slag in a Circular Economy Framework

**DOI:** 10.3390/ma17163945

**Published:** 2024-08-08

**Authors:** Anja Terzić, Jovica Stojanović, Vladimir Jovanović, Dejan Todorović, Miroslav Sokić, Dragan Bojović, Dragan Radulović

**Affiliations:** 1 Institute for Testing of Materials, Bulevar Vojvode Mišića 43, 11000 Belgrade, Serbia; dragan.bojovic@institutims.rs; 2 Institute for Technology of Nuclear and Other Mineral Raw Materials, Franchet d’Esperey 86, 11000 Belgrade, Serbia; j.stojanovic@itnms.ac.rs (J.S.); v.jovanovic@itnms.ac.rs (V.J.); d.todorovic@itnms.ac.rs (D.T.); m.sokic@itnms.ac.rs (M.S.); d.radulovic@itnms.ac.rs (D.R.)

**Keywords:** waste resources, metallurgy, microstructural analysis, critical minerals, non-ferrous metals, zero waste

## Abstract

The presented work offers an innovative process scheme for valorizing Pb-Zn slag, which involves crushing, grinding, and separation techniques to concentrate valuable components (non-ferrous metals). This methodology could have a significant impact on the global beneficiation of metallurgical slags since it is significantly more simple, environmentally friendly, and cost-effective than standard pyro- and hydrometallurgical procedures. According to previous physicochemical and mineralogical studies, Pb-Zn slag is a valuable secondary raw material. This inhomogeneous technogenic resource contains substantial amounts of non-ferrous metals (Pb, Zn, Cu, and Ag). However, laboratory tests have indicated that the Pb-Zn slag contains highly uneven amounts of valuable metals, ranging from several g/ton to tens of g/ton. The main issue is that traditional metallurgical procedures for releasing beneficial elements are not commercially viable since the elements are “trapped” within the amorphous aluminosilicates or intergrowths of alloy grains and glassy phases. Gravity concentration (Wilfley 13 shaking table) and magnetic separation (Davis separator and disk separator) were used to obtain the final concentrate following comminution and grindability testing. The gravity concentration proved more effective. Namely, magnetic separators could not process nor adequately separate beneficial non-ferrous elements because they were merged together with iron-bearing minerals and aluminosilicates in amorphous Pb-Zn slag grains. With the gravity concentration approach, 12.99% of the processed slag belonged to ∆T fraction (concentration of non-ferrous metal alloys), while remaining 87% corresponded to the tailings fraction (∆L). The total amounts of recovered Pb, Zn, Cu, and Ag from ∆T and ∆L fractions were 5.28%, 6.69%, 0.58%, and 76.12 ppm and 1.22%, 6.05%, 0.43%, and 15.26 ppm, respectively. This streamlined approach to valorizing Pb-Zn slag can reduce the need for hazardous chemicals used in hydrometallurgical refinement operations, as well as the extremely high temperatures required for pyrometallurgical processing. This is the first study to investigate the viability of this novel methodology, which involves the direct examinations of the Pb-Zn slag feed with various alternative technologies for separation and concentration. After extracting the valuable metals, the amorphous aluminosilicate part of the Pb-Zn slag can be reapplied as an alternative raw material in the building sector, adding to the circularity of the suggested approach.

## 1. Introduction

A critical resource is, by definition, a raw material that can only be used by at most one process at any one time. Critical minerals are considered as critical resources. They are commodities found in a wide range of ore deposits that are critical to the economic or national security of individual nations and are susceptible to supply disruption [1,2]. What is considered a critical resource depends on the branch of industry, the cost of processing, and the potential of returning the resource to the same or another process [3]. Critical minerals, including Cu, Li, Ni, Co, and REE, are essential components for many of the rapidly developing renewable energy technologies, including electric vehicles, power networks, and wind turbines. Still, a large number of ores and minerals can be considered critical or at least essential. Most material resources have varying levels of risk in terms of supply interruption, vulnerability, and environmental and social repercussions [4]. All natural resources can become critical at some point. Therefore, developing critical mineral projects for a decarbonized future is a key asset of contemporary scientific and industrial practice [5,6]. Lead and zinc are non-ferrous metals that are most commonly used in different industrial fields, after copper and aluminum [7]. The US Geological Survey has already included Zn on its critical minerals list [8]. In the ongoing decade, the amount of Zn mined worldwide is estimated at 13 million tons. The Zn production carbon footprint, as determined by the GHG Protocol Standard method, is 0.93 tons CO_2_e per ton of Zn [9,10]. Total estimated global Pb reserves are 85 million tons; however, the annual amount of mined Pb is 4.49 million tons worldwide [11,12]. Lead has a much smaller carbon footprint than zinc, ranging from 30 to 76 kg CO_2_/kg of Pb [13,14].

High quantities of non-ferrous metals tend to accumulate in waste products generated during the smelting of metal ores [15]. Environmental concerns, i.e., huge amounts of waste generation, as well as gaseous and particulate emissions (SO_2_, CO_2_, CO, Hg, As-, Sb-, Se-, and Te-oxides) have led to a shift toward hydrometallurgy regarding the refinement of non-ferrous metals. However, pyrometallurgical processing still accounts for 10% to 20% of Zn and Pb total output [16]. Zn and Pb can be obtained from smelter slags at volumes of 70 and 7 kt/y, respectively [17]. The great majority of these waste materials (slags) are disposed of, temporarily kept in industrial solids storage facilities or in open landfills due to a lack of commercially viable technologies for their valorization. Pb and Zn are both mostly present in amorphous phases, from which extraction can be challenging especially with pyro- and hydrometallurgical procedures due to the huge environmental risk (previously mentioned gaseous and particulate emissions, pressure leaching, chloride leaching, and biologically assisted leaching). Current methods of treating metallurgical slags, including reprocessing, desulfurization, backfilling, and restoration, are not commonly employed due to their high cost and/or limited efficiency [18,19]. Solid waste in remote rural areas, especially those close to abandoned mining sites or smelters, has received far less attention because the local authority cannot afford the expenses of waste handling. Consequently, the development of waste treatment technologies that are technically, environmentally, and economically feasible becomes imperative. The Pb-Zn slag has several possible uses due to its specific mineralogical characteristics, namely, there is economic potential for the extraction of valuable metals, including Ag, Pb, Zn, and Cu. In order to produce metal concentrates from their collected concentrates, techniques including gravity concentration and magnetic separation are being constantly developed [20]. Upon the extraction of valuable elements, the rest of technogenic material can be reapplied in civil engineering due to its aluminosilicate origin [21,22,23]. Using Pb-Zn slags to produce building materials is a feasible and profitable approach to recycling them, especially if the end-products are produced and sold locally, which aligns with the Sustainable Development Goals (SDGs) put forth by the UN in the 2030 Agenda. The metallurgy and mining industries are under pressure to develop sustainable technologies to satisfy SDGs [24,25,26].

The topic of revalorization and extracting valuable elements by magnetic separation and/or gravity concentration is a subject of numerous investigations. Each raw material (slag) is different and depends on its origin, processing methodology, and environmental conditions upon landfilling; therefore, every new defined procedure for refinement represents an entirely new case study, just as this research is completely different and unique in comparison to work by other authors. For instance, the recovery of the minor elements (V, Cr, Mn, Co, Ba, La, Ce, and Fe) contained in waste landfills can be conducted via gravity and magnetic force sorting. The chemical morphology of each element directly influences the processing methodology. A high resulting metal concentration can be obtained with the gravity sorting process when the specific gravity of the heavy liquid is 2.5–2.75 g/cm^3^. Magnetic force sorting can efficiently separate metals and non-metals and is effective as a pretreatment for a more advanced concentration stage [27]. Gravity separators are constantly being improved and upgraded by developing mathematical models employed in novel experiments such as three-phase separation [28], thereby even more efficient results coming from this concentration technique can be expected in the near future. Magnetic separation can be used in iron enrichment from Zn-Pb-bearing refractory iron ore (47.04% Fe, 0.39% Pb, and 0.30% Zn). Fe is successfully extracted by magnetic separation, but elevated temperatures decrease Zn and Pb contents in concentrate [29], which also categorizes magnetic separation as pre-treatment. Supergravity can be used for recovery of boron from boron-bearing Fe concentrate (painite ore tailings) through mineral phase transformation and low-temperature (1523 K) separation. The boron is recovered from the B-rich slag with a high recovery of 98.24%. This method efficiently improved the boron recovery as well as the activity of B-rich slag for sustainable utilization [30]. Supergravity can also be employed as an efficient method for the purification of Cu alloys, the removal of trace impurities, and the recycling of valuable components from secondary sources (waste-stained copper wire) [31]. Rare earth mineral deposits are typically processed using several different unit operations, including flotation, gravity, magnetic, and electrostatic separation techniques, but the two of the most important beneficiation techniques for RE minerals are gravity and magnetic separation [32,33], which coincide with techniques selected for this research. Furthermore, Pb-Zn mine tailings are reprocessed using centrifugal dense media separation (DMS), which allows the high recovery of cerussite (PbCO_3_) and smithsonite (ZnCO_3_). Also, DMS successfully depollutes the studied mine tailings [34]. Gravity separation is used in enhanced desliming and as a precursor to flotation in the upgrading of cassiterite from tailings [35]. Magnetic and gravity separation are employed in transforming iron ore processing by simplifying the comminution and replacing reverse flotation [36]. Thereby, both of these procedures, i.e., magnetic and gravity separation, are considered crucial steps in the beneficiation of valuable elements from waste resources.

The goal of this study is to establish a novel and unique process circuit for the valorization of historic Pb-Zn slag from “Topilnica” Veles (Northern Macedonia). Pb-Zn slag represents a potentially valuable secondary raw material with significant contents of non-ferrous metals (Pb, Zn, Cu, and Ag), but the main problem is that the elements are “trapped” within amorphous intergrowths of alloy grains, minerals, and aluminosilicates. Standard pyro- and hydrometallurgical procedures for element liberation are not environmentally sustainable according to the Green Agenda, as there is risk of gaseous and particulate emissions, pressure-, chloride-, or biologically assisted leaching. Magnetic separation using Davis and disk separators, as well as gravity concentration using a Wilfley 13 shaking table, are expected to be demonstrated as alternatives to traditional metallurgical operations for non-ferrous element valorization. The cost-effectiveness, environmental safety, and viability of these techniques are ascertained because, after extracting the valuable metals, the amorphous aluminosilicate part of the Pb-Zn slag will be reapplied as an alternative raw material in the building industry, contributing to the circularity of the proposed procedure.

## 2. Materials and Methods

### 2.1. Characterization of Pb-Zn Slag

The artificial (secondary) mineral raw material, i.e., Pb-Zn smelter slag from the Topilnica Veles, North Macedonia (KEPS MONT GROUP, Skopje, North Macedonia), was analyzed in this study. Currently, there are 2,000,000 t of this historic Pb-Zn slag located at the landfill near the smelter. The preliminary analyses of this raw material were conducted in our previous work [37]. The chemical analysis of the Pb-Zn slag detected: SiO_2_ (17.4%), Al_2_O_3_ (7.4%), CaO (12.3%), MgO (2.1%), Fe_2_O_3_ (47.7%), Pb (2.3%), Zn (7.1%), S (2.1%), and Ag (27.5 ppm) [37]. The slag with this chemical composition has a realistic potential for further refining, with the goal of extracting valuable elements and reusing the remaining raw material in civil engineering (i.e., construction materials such as mortar or concrete) in accordance with Circular Economy and Zero Waste principles.

The composition of this artificial raw material is complex, consisting of amorphous and mineral phases such as wurtzite ((Zn,Fe)S), sphalerite (ZnS), galena (PbS), cerussite (PbCO_3_), akermanite ((Ca_2_Mg[Si_2_O_7_]), wüstite (FeO), monticellite (CaMgSiO_4_), franklinite (ZnFe_2_O_4_), and zincite (ZnO) [37]. Chemical analysis, X-ray diffraction (XRD), and scanning electron microscopy (SEM) with an energy dispersive X-ray spectrometer (EDS) revealed the presence of a variety of inclusions containing lead alloys, zinc alloys, elemental silver, copper, and iron. Melted silicates, mixed aluminosilicates, and Fe-Mn-Zn spinels are most likely responsible for the occurrence of the amorphous phase. Wüstite, as a source of iron, which emerged as skeletal inclusions in the glassy matrix, was far less abundant [37]. According to SEM/EDS, lead and zinc alloys contained a high concentration of Cu. Alloys appeared predominantly as inclusions or as simple to complex conglomerates with a glassy matrix of elemental iron or wüstite. The majority of alloy grains were up to 100 µm in diameter, although some were bigger (up to 300 µm). Alloy grains larger than 100 µm were free or in the form of simple conglomerates [37]. The presence of visible and/or “invisible” (i.e., structural) silver was not detected in Zn-alloys. Grains corresponding to Pb-alloys appeared in the form of spheres, and unlike Zn-alloys, they comprised both visible and structural Ag [37]. Silver was oval-shaped or in the form of small wires of up to 5 µm in size. Elemental silver and copper were found in both the glassy matrix and wüstite as tiny inclusions of 2–3 µm and 7–8 µm, respectively [37]. Elemental silver can also be found in the crevices of host grains [38]. Iron (in elemental form) was mostly found as wüstite, magnetite, hematite, troilite, and spinel, but in considerably smaller levels in pyrite and arsenopyrite [37,39]. 

The mineralogical and microstructural examination revealed that the investigated raw material is extremely complex. Therefore, for it to be reused, Pb-Zn slag has to be thoroughly examined to determine what component may be valorized and how this can be accomplished. This type of secondary raw material is unique to each smelter; there is no laboratory or factory procedure for treating it, hence there are no starting points for further refinement and valorization. As a result, it was decided to follow the standard procedure for processing and refining technogenic raw materials, to record all obtained outputs, and to include analyses at each stage of the procedure to demonstrate the significance of the preparation and refinement process, as well as methods for concentrating useful components into commercial products.

### 2.2. The Preparation of Input Slag Sample and Experimental Plan for Technological Testing

**Figure 1:**
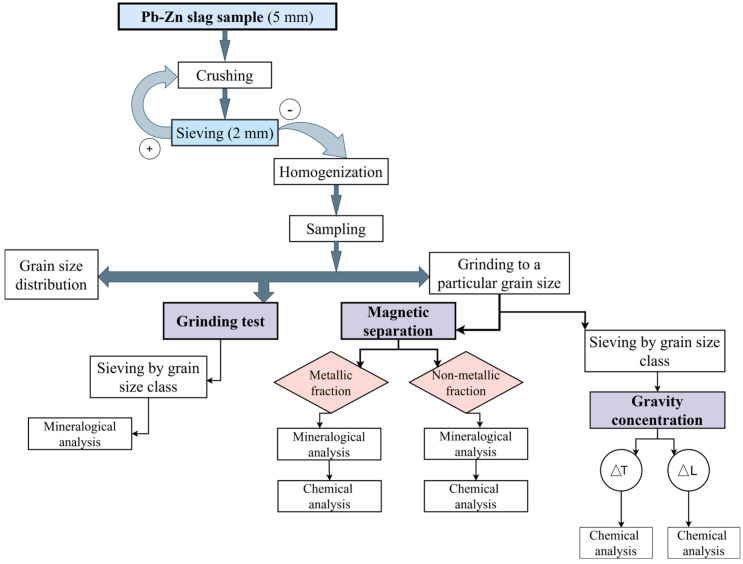
The experimental plan of Pb-Zn slag treatment.

The input Pb-Zn slag sample weighed 50 kg and had an upper grain size of 5 mm. The preparation of the sample (crushing, sieving, homogenization, and sampling) for further technological testing was performed in Phase 1 (Figure 1, upper side of the scheme). Primary crushing was conducted on a laboratory jaw crusher and subsequently on a roll crusher. Crushing using a jaw and roll crushers revealed that the raw material is exceedingly hard and difficult to break, and that during crushing, it frequently takes on needle-like and elongated shapes.

The input slag sample was crushed or milled to a maximum size of 2 mm for further sieving tests. Sieves with a 2 mm opening were used. The residue on sieves was returned to the “crushing step”. Grain-size classes below 2 mm were submitted to homogenization and sampling. The samples of 1 kg mass were employed in further phases.

Phase 2 (Figure 1, lower left side of the scheme) included grindability tests and the determination of the grain size distribution. Grinding was conveyed on a laboratory batch ball mill “Denver” [40]. Grindability tests were performed as part of the technological preparation to find the ideal conditions for separating and releasing beneficial components from inert and harmful ones contained in the Pb-Zn slag.

Phase 3 (Figure 1, lower right side of the scheme) of the experiment included determining the likelihood of useful metal concentrations (Pb, Zn, Cu, and Ag) and selecting the optimal approach. This phase focused on methods for separation and concentration (magnetic separation and gravity concentration) and the assessment of the acquisition of useful components in differently processed products.

The experimental plan is illustrated schematically (Figure 1). In Figure 1, ∆L is the tailings fraction and ∆T is the concentration of non-ferrous metal alloys obtained upon conducting gravity concentration.

### 2.3. Determination of the Grain-Size Distribution of the Pb-Zn Slag (Starting Sample)

**Figure 2:**
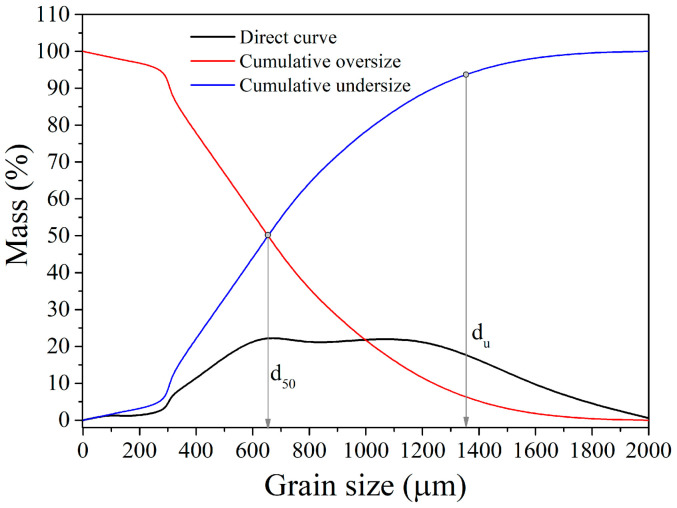
Grain size distribution of the starting Pb-Zn slag sample.

The grain size distribution of the Pb-Zn slag sample (starting sample) was determined following grinding on a Denver-type laboratory batch mill. The upper grain size of the feed was 2 mm (2000 µm). The granulometry of the resulting Pb-Zn slag sample was determined using Tyler’s series of sieves [41], with the last sieve in the sequence having a 100 µm opening. All the oversize masses, starting with the first sieve and moving towards the last sieve in the set, were weighted, and the cumulative undersize and the direct curve were calculated and are illustrated in Figure 2.

The obtained average grain diameter of the Pb-Zn slag sample is d_50_ = 650 µm, while the upper limit of grain size (d_u_) is 1310 µm.

### 2.4. Grindability Test

A grindability test analyzes a material’s resistance to ball milling. This test is helpful for calculating and comparing the Bond Work Index (BWI) of various ores and ore-like materials. Grindability is normally measured by determining the number of revolutions in a pulverizer that are required to achieve a given size reduction. This is an indirect indication of how much work is required to reduce that size. Two types of grindability testing (GT) were conducted in this experiment: (1) wet grinding (WGT) and (2) dry grinding (DGT). WGT is usually more effective than DGT. When it comes to ores or ore-like materials, wet grinding has three advantages over dry grinding: (1) precision—wet grinding frequently results in finer particle size; (2) adaptability—WGT may be applied to a wide range of materials, including those that create heat or become reactive when ground; and (3) safety—less dust equals a safer working environment. Furthermore, WGT is more energy efficient than DGT, requiring up to 30% less power to operate a wet mill. Over time, the energy savings can accumulate and offset part of the expense of the additional drying stage. Therefore, the DGT was conducted once for a duration of −20 min, which is the median milling time for the first stage of WGT. All adopted milling times were results of the assessment of initial testing trials. Keeping in mind that DGT is energetically less efficient, this method was conducted only for comparison of the results. The WGT was initially planned to be conducted in two stages, i.e., with 60% and 70% of solid phase (SP), respectively. However, during the test with 60% of SP, it was observed that an excessive amount of water appeared in the mill. Pb-Zn slag did not behave like an ore or ore-like material. Namely, grinding an ore with a high content of silicates and a low content of metallic minerals results in pulverized matter in the form of a pulp that envelops the grinding tools like a film, which helps the comminution. Contrary to the previously described scenario, in the 60% SP test, the pulp was not formed. Instead, the excess water remained in one part of the mill, which enabled further testing. Therefore, only the test with the 70% SP was undertaken.

The GT identifies the ideal grinding duration and slag’s fineness (an optimal grain size class) that enables the maximal release of valuable components (metal minerals). Two experimental sets were carried out in the “Denver” laboratory batch mill (the mass per sample was 1 kg and the maximal grain diameter was 2 mm). Three grinding times (t) were employed in Experimental Set No. 1 (ES-1): 14 min, 20 min, and 26 min. Grinding times for Experimental Set No. 2 (ES-2) were: 32 min, 38 min, and 44 min. The volume ratio between powder/liquid/milling medium was kept at 1:1:1. Following the grinding procedure, all the obtained products underwent visual qualitative analysis with an optical microscope. The fineness of the powder was determined by sieving (set of sieves with openings: 150 µm, 100 µm, and 25 µm in ES-1, and 100 µm, 75 µm, 53 µm, 37 µm, and 25 µm in ES-2).

### 2.5. Magnetic Separation 

Magnetic separation (MS) tests were carried out on Pb-Zn slag with a size class of −0.1 + 0.00 mm (−100 + 0.0 µm). Commercial magnetic separators are continuous-process devices which use a moving stream of particles that enter and exit the magnetic field to perform separation. Particle speed throughout the field must be precisely regulated, which usually means using free fall as a form of feeding [42]. The MS was performed using two devices: a Davis magnetic separator (“wet” method) and a magnetic separator with a disk (“dry” method). The sample for MS was prepared by combining the grain size classes obtained during GT with diameter limits of −100 + 0.0 µm. This sample was separated into two parts: one for the MS test on the Davis separator and the other for the disk separator. In the Davis separator, the separation tube is placed between the poles of a powerful electromagnet at an angle of 45° (configurable). The tube is agitated back and forth and rotated by a motorized system. Magnetic particles are collected inside the tube in the zone of high magnetism, while non-magnetic minerals are washed away [43]. The Davis separator with a maximal magnetic induction of 700 gauss = 0.07 T was used in the experiment. A magnetic disc separator uses electromagnetic forces to separate materials with different magnetic susceptibilities. This separator typically consists of three high-intensity electromagnetic disks, each placed at a different height from the feed conveyor [44,45]. The magnetic separator with a disk used in this experiment had a maximum current of I = 1.8 A and maximal magnetic induction of 11,000 gauss = 1.1 T. 

### 2.6. Gravity Concentration 

The gravity concentration (GC) was performed on Pb-Zn slag with a size class of −0.1 + 0.00 mm (−100 + 0.0 µm). Similar to the MS experiment, the raw material’s size class was assessed using the mineralogical properties of Pb-Zn slag, structural–textural properties, degree of cohesiveness (i.e., freedom), and concentration criteria. Namely, because more than 85% of the usable components are free in grain classes with a diameter of less than 100 µm, GC was performed on a sample of this size. The grain size classes used as an input for the GC (obtained by wet sieving) are −100 + 75 µm; −75 + 53 µm; −53 + 37 µm; −37 + 25 µm; and −25 + 0.0 µm.

**Figure 3:**
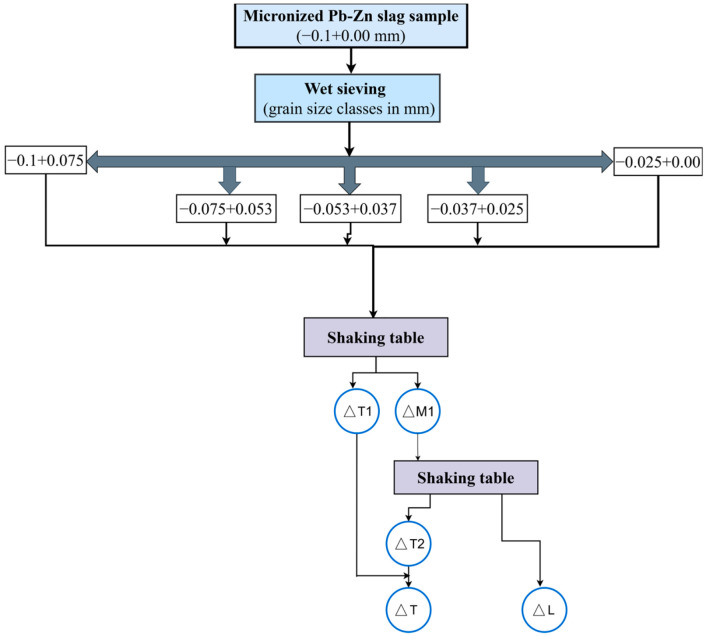
Scheme for preparing and separating Pb-Zn slag samples using the GC method.

The GC of the individual grain size classes was carried out on a Wilfley 13 shaking table. The function of the Wilfley table is to separate two or more mixed materials based on their density. This device works by flowing items across a level surface, i.e., the table, using water. The water serves as a medium of separation [46,47]. The table’s working area has been optimized to handle mud and finely ground raw materials. The Wilfley shaking table has proven to be a very effective tool for concentrating small classes by gravity in a thin layer of water. Based on theory and experience, the gravity concentration procedure was designed to yield the definitive products (∆T and ∆L) after double purification. ∆L is the tailings fraction and ∆T is a concentration of non-ferrous metal alloys. Figure 3 depicts an established procedure for the sample preparation and testing via GC. 

In Figure 3, ∆T1 is a concentration of non-ferrous metal alloys after initial treatment on shaking table (first step of GC procedure), while ∆M1 is remaining mass of the sample which subjected to the second step of the GC procedure. ∆T2 is a concentration of non-ferrous metal alloys after second treatment on shaking table (second step of the GC procedure). ∆L is total tailings fraction and ∆T is total concentration of non-ferrous metal alloys that exits GC treatment. 

### 2.7. Instrumental Analyses

Chemical analysis, i.e., the identification and quantification of selected chemical elements was conducted using the atomic absorption spectroscopy (AAS) technique on a PinAAcle 900 Perkin Elmer instrument (PerkinElmer, Waltham, MA, USA). AAS has a high degree of accuracy, with results typically ranging from 0.5 to 5%. It is a highly sensitive test procedure that can measure as low as parts per billion (g/kg) depending on the material under test. The instrument is a flame-only device with a real double-beam design for quick startup and long-term reliability. It has fiber optics to maximize light flow for greater detection limits, an eight-lamp mount, and automated flame and burner assembly optimization for increased production. Pulverized slag samples (d_50_ < 63 μm) were used in the analyses. Samples were dried at 105 °C before pulverization in a planetary ball mill.

Microscopic analysis: medium-sized grain classes (about −2.38 + 0.00 mm) of Pb-Zn slag were placed in Plexiglas plates for optical microscope recording. The preparation area was 2.2 cm^2^. The quartering procedure maintained the authenticity of the starting grain mixture. The visual examination of the samples helped in revealing the range of mineral phases found in the original slag sample. The analysis was performed using Carl Zeiss-Jena’s JENAPOL-U polarizing microscope for transmitted and reflected light (ZEISS, Reutlingen, Germany), as well as a measuring tool. The samples were recorded using an Axiocam 105 color camera and the Carl Zeiss AxioVision SE64 Rel. v4.9.1 software package, which included the multiphase module.

Scanning electron microscopy with an energy dispersive X-ray spectrometer (SEM/EDS): The Pb-Zn slag sample was carbon-coated (20 nm layer, density 2.25 g/cm^3^). The analysis was performed on the JEOL JSM-6610LV SEM (JEOL, Akishima, Tokyo, Japan). The magnification range for this device is ×5 to ×300,000. The electron source was a W wire (LaB 6). The voltage ranged from 0.3 to 30 kV. The instrument employs a vacuum system consisting of a rotary pump and turbomolecular pump (included in the microscope’s basic configuration), an ion pump (for LaB6), and a rotary pump for low vacuum (10–270 Pa). The following detectors were used: SE, BSE, CL, and EDS. The detection limit for elements Z ≥ 5 is approximately 0.1 wt.%, with a resolution of 126 eV. The sample chamber has five axes of movement: X, Y, Z, T-tilt, and R-rotation; the maximum sample size is 20 cm (width), 8 cm (height), and 1 kg. Two infrared cameras were included. The microanalysis standards included 64 natural minerals and synthetic chemicals. The LEICA SCD005 (Leica Microsystems, Wetzlar, Germany) is used to coat samples with gold (Au) or carbon. An ultrasonic bath, binoculars, and other equipment were also present. A voltage of 20 kV and an extinction duration of 50 s were used. The lower limit of EDX sensitivity was around 0.3%.

## 3. Results

### 3.1. Determination of Grindability 

A grindability test was employed to identify the conditions (grinding time and grain size class) for separating useful components in Pb-Zn slag from inert or hazardous ones. The mechanical processing of slag and other similar technogenic raw materials containing a certain percentage of non-ferrous metals is a field lacking in technological know-how and experience. The experiment was conceptualized based on previous experience in grinding hard metallic ores with high aluminosilicate concentrations. Wet grinding was used for a variety of reasons: (1) it complements the subsequent concentration procedures (wet magnetic separation and wet gravity concentration), and (2) it is up to four times more efficient than dry grinding (uses less energy for the same grinding action).

Pb-Zn slag contains valuable minerals (wurtzite, sphalerite, galena, cerussite, akermanite, wüstite, monticellite, franklinite, and zincite [37]) corresponding to metal alloys present in the form of complex intergrowths. The amorphous phase (glassy tailings) of aluminosilicates, silicates, spinel, and mixed spinel–silicate composition, as previously assumed, is the predominant phase in observed slag samples [37]). The amorphous phase is responsible for the extremely high hardness of Pb-Zn slag. Upon grinding (short grinding periods), non-metallic mineral phases (crushed parts of the glassy matrix) are expected to appear in oversize classes (a residue on a sieve). Metal minerals are the “softer” part of the slag’s complex composition; therefore, they are expected to be in undersize classes. 

The initial sieve test design (ES-1) included the following projected sieve openings: (1) sieves with a 150 µm opening to separate coarser grain sizes with no useful components (i.e., crushed glassy phase); (2) sieves with 150 µm and 100 µm openings to conduct “pre-concentration” of useful minerals (softer metal minerals and/or their complex intergrowths); and (3) sieves with 25 µm opening to separate the finest class in order to determine if metallic components (lead–copper and zinc–copper intergrowths) have transitioned into it while grinding.

**Figure 4:**
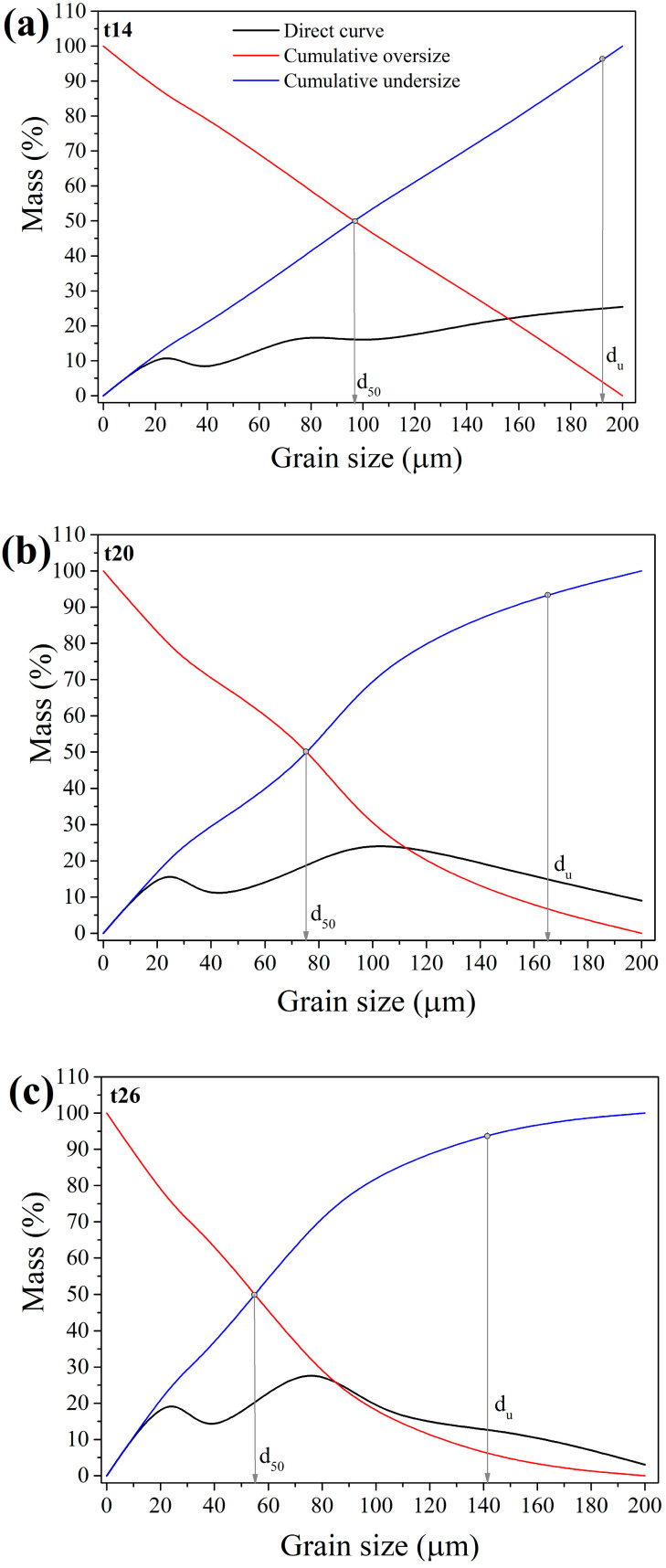
Grain size distribution for ES-1: (**a**) t = 14 min; (**b**) t = 20 min; and (**c**) t = 26 min.

The purpose of these tests was to detect the grain size classes in which non-useful tailings are concentrated so they could be eliminated from further processing in order to decrease energy consumption during milling. Furthermore, the detection of the exact particle size of metallic components is important, so their migration into the fine-size class is prevented because concentrating them at that point becomes challenging. Figure 4 shows grain size distribution diagrams for ES-1 for intervals (t) of 14, 20, and 26 min (i.e., GT-1, GT-2, and GT-3). 

As shown in the graphs (Figure 4), longer grinding periods resulted in a material with a smaller mean grain diameter (d_50_) and upper grain limit (d_u_): t = 14 min, d_50_ = 97.5 µm, d_u_ = 193.3 µm; t = 20 min, d_50_ = 76.3 µm, d_u_ = 165.7 µm; and t = 26 min, d_50_ = 55.7 µm, d_u_ = 142.5 µm.

In the first GT (t = 14 min), mineralogical analysis revealed that the coarsest grain class (+150 µm) contained fused vitreous phase and metal alloy grains. Analysis was conducted by the visual examination of the grain mixture obtained as oversize on a 150 µm sieve. The sample was observed by an optical microscope (described in Section 2.7). This class accounts for 25.41% of the overall micronized product in GT-1. Conglomerates and intergrowths were found in the −150 + 100 µm class. In the finest grain size class (−25 + 0.0 µm) of the slag sample, alloy grains and tailings were indistinguishable. The finest class accounted for 14.83% of the total mass used in the GT-1.

With an extended grinding in the second GT (t = 20 min), mineralogical analysis revealed, same as in the GT-1, that the coarsest class (+150 µm) is composed of fused glassy phases and metal alloy grains. The fused inclusions and intergrowths were also evident in the finer class (−150 + 100 µm). The number of free alloy grains increased. In the finest class (−25 + 0.0 µm), alloy grains were found to glow under the microscope’s reflected light (i.e., they are distinguishable). A longer grinding period of 20 min resulted in a higher mass share of the fine grain size class (21.39%), which is roughly 6.5% higher than GT-1.

In the third GT (t = 26 min), the mineralogical analysis revealed that the coarsest grain size class (+150 µm) contains fused glassy phases and metal alloy grains, as well as very large free metal alloy grains. The share of the +150 µm class in the total micronized product is rather small (3.06%). The middle class (−150 + 100 µm) consisted of more free alloy grains than fused grains. Alloy particles were identifiable in the −25 + 0.0 µm class. The grinding period of 26 min produced the highest mass share of the finest grain size class, its participation in the GT-3 being 26.98%. That is a mass increase of approximately 5.6% over the GT-2 and 12.15% over the GT-1. The obtained results of the mineralogical analysis of the GT-3 show that the coarse class (+150 µm), despite its small mass share of 3.06% in the total amount of micronized product, cannot be rejected as the class in which tailings are concentrated solely due to the presence of large alloy grains.

**Figure 5:**
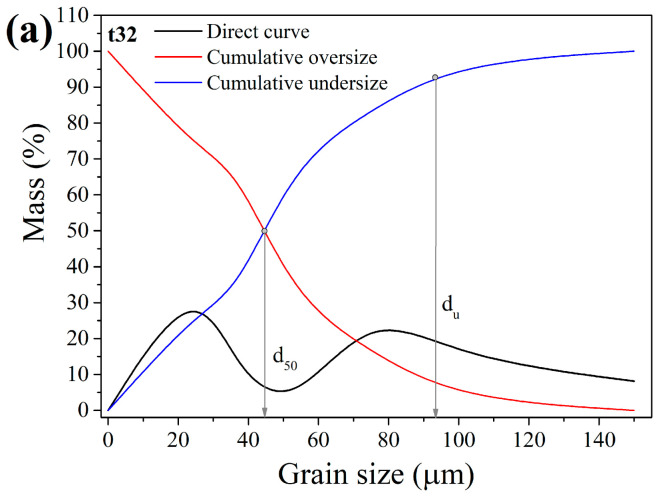

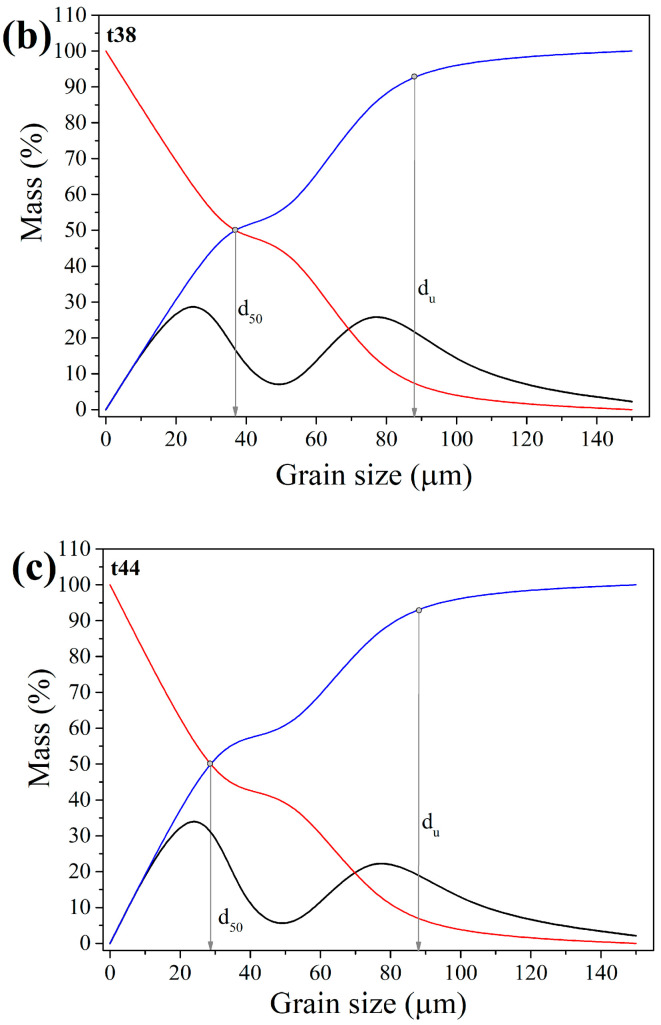
Grain size distribution for ES-2: (**a**) t = 32 min; (**b**) t= 38 min; and (**c**) t = 44 min.

The second sieve test design (ES-2) included the following projected sieve openings: i.e., grain size classes: −100 + 75 µm; −75 + 53 µm; −53 + 37 µm; −37 + 25 µm; and −25 + 0.0 µm. Figure 5 shows grain size distribution diagrams for ES-2 intervals (t) of 32, 38, and 44 min (i.e., GT-4, GT-5, and GT-6). These GTs did not contain a +150 µm grain size class as a result of the extended milling. The coarsest grain size class found in these samples was −150 + 100 µm.

Grinding for a longer period reduced the mean grain diameter (d_50_) and upper grain limit (d_u_) of the treated Pb-Zn slag (Figure 5): t = 32 min, d_50_ = 44.2 µm, d_u_ = 93.4 µm; t = 38 min, d_50_ = 37.2 µm, d_u_ = 89.1 µm; and t = 44 min, d_50_ = 28.9 µm, d_u_ = 87.2 µm.

In the GT-4 (t = 32 min), the −150 + 100 µm class accounted for only 8.12% of the total mass. This class contains fewer coalescing alloys and less amorphous phases, as well as more free alloy grains. The rest of the observed classes (−100 + 75 µm; −75 + 53 µm; −53 + 37 µm; −37 + 25 µm; and −25 + 0.0 µm) comprised free alloy grains, vitreous phase, and silicates. The finest class (−25 + 0.0 µm) contained small metal grains up to 10 µm in size. Although the Pb-Zn slag sample was ground for 32 min, there are free alloy grains and a small amount of coalesced mineral grains in the coarsest class (−150 + 100 µm); therefore, this class cannot be rejected as a class in which tailings are concentrated. The mass percentage of the finest class (−25 + 0.0 µm) increased to 38.45%, which is an 11.47% increase over the GT-3 (t = 26 min).

The coarsest class (150 + 100 µm) accounted for only 2.22% of the total mass after the GT-5 (t = 38 min). There were more free alloy grains, a few alloy fusions, and amorphous phases in this class. All other classes contained free alloy grains, a glassy phase, and silicates. Spinel was found in the −53 + 37 µm class. It was in needle-like form, at up to 150 µm in length and 30–35 µm in width. This might be the outcome of the slag grains breaking into long, needle-like shapes due to the mechanical crushing [48,49]. The −25 + 0.0 µm class contained a considerable number of metal grains, about 10 μm in size. Even with the extra grinding (38 min total), the mass percentage of this class in the entire mass increased only marginally to 38.96%. This shows a slight increase of 0.51% over the GT-4, when the mass share of the finest class was 38.45%.

Even though the mass share of the coarsest class (−150 + 100 µm) was only 2.12% in the GT-6 (t = 44 min), free alloy grains and silicates were still present. The analyzed grain size classes in the GT-6 were identical to the two previous GTs. Free alloy grains, the glassy phase, and silicates were found in all classes. As in the previous test, a needle-shaped spinel was found in the −53 + 37 µm class. Very fine metal grains were found in the −25 + 0.0 µm class. This class’s mass participation ascended to 48.20% as the grinding time increased (t = 44 min), which is a 9.24% increase from the GT-5.

**Figure 6:**
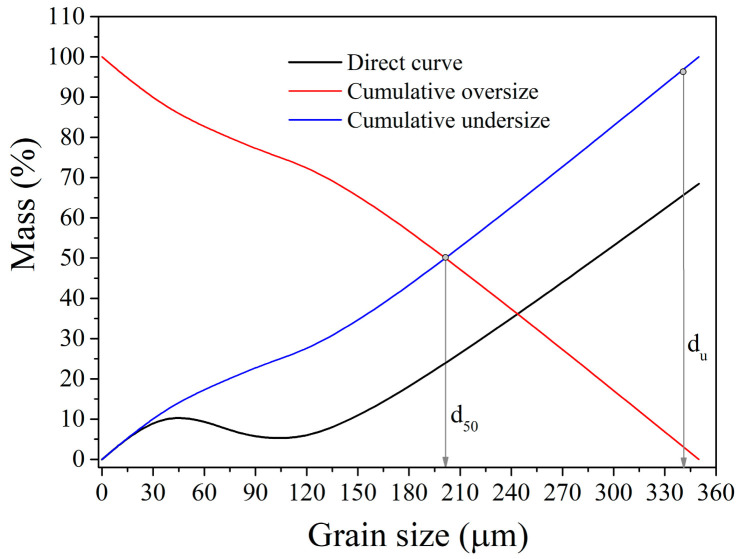
Grain size distribution of the Pb-Zn slag grain mixture after dry grinding.

Figure 6 shows the grain size distribution of the Pb-Zn slag grain mixture obtained after dry grinding for 20 min. The dry-grinded mixture had a mean grain diameter of 206.1 µm and an upper grain size of 335.2 µm. Thus, wet grinding (t = 20 min) with the same energy and time input outperformed dry grinding of the identical material.

The GT-1–3 results indicate that no test, not even with the shortest or longest grinding duration, can exclude the coarsest class (+150 µm), in which tailings were expected to be concentrated. The microscopic observation of this class revealed free alloy grains and fusions of glassy phase and alloys; therefore, it cannot be described as a tailings-concentrated class. The coarsest class in the GT-4–6 was −150 + 100 µm, which also could not be excluded from GTs as a class that contains concentrated tailings solely. Namely, this class contained grains of metal alloys and conglomerates; hence, eliminating it would result in a loss of metal utilizability. 

The GTs supplied fundamental information on the grinding process and the release of usable components from Pb-Zn slag. It was determined that most of the free utilizable components are present in the −100 + 0.0 µm class. Therefore, this class was further used in magnetic separation and gravity concentration tests, which are methods for successfully separating non-ferrous metal alloys and minerals into one product while separating tailings into another.

### 3.2. Magnetic Separation of Pb-Zn Slag

After GTs, the Pb-Zn slag was subjected to a magnetic separation test to further reduce inefficient components. MS removes iron-containing magnetic components from the sample and is expected to result in final products (concentrates) with a higher metal content (relative to the input) and thereby improved utilizability. “Utilizability” is percentage of utilizable metal from its concentrate. The aim of this technological process (MS) is to achieve the highest possible utilization of metals and the highest degree of concentration of metals in concentrates. “Wet” and “dry” separation procedures were tested on a Pb-Zn slag sample of −100 + 0.0 µm grain size.

#### 3.2.1. Magnetic Separation of Pb-Zn Slag by Davis Separator

**Figure 7:**
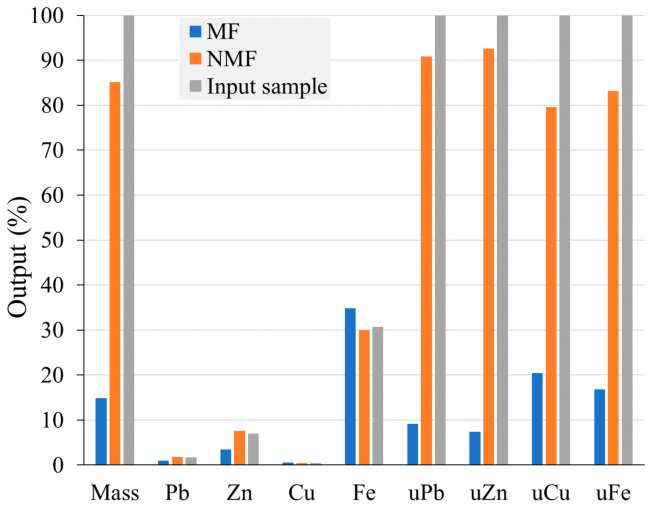
Magnetic separation balance of Pb-Zn slag after application of Davis separator.

The Pb-Zn slag magnetic separation experiment was conducted using a Davis separator, which divided the sample into its magnetic fraction (MF) and non-magnetic fraction (NMF). Both fractions were dried and subjected to chemical analysis via AAS. The results are reported in the form of a material balance in Figure 7. Recovery per element is calculated from the mass of starting sample (input, NMF and MF, i.e., the initial stacks in Figure 7). 

The analysis of the results of MS performed on the Davis separator revealed that the MF and NMF were not adequately separated. The MF accounted for 14.81% and the NMF 85.19% of the total processed slag’s mass. A considerable amount of non-ferrous metals that have been separated by applying a low magnetic field are present in the MF: 1.03% lead, 3.48% zinc, and 0.62% copper (Figure 7). The amount of Cu in MF is 38% higher than that of the input (0.45%). Non-ferrous metals are known to be non-magnetic; therefore, their presence in the MF is uncommon, especially if a low magnetic field is employed for separation, which should only separate strongly magnetic components. The reason for the presence of Zn in the MF could be the mineral franklinite (ZnFe_2_O_4_). Theoretically, franklinite is composed of 16.59% Zn and 37.78% Fe [50]. Thereby, the presence of Zn in the MF can be explained either by its association with franklinite or amorphous formations in the slag that are chemically similar to franklinite. However, this explanation is not feasible for Pb or Cu. 

Due to the small mass share of the MF (14.81%), the utilizability (marked with “u” in Figure 7) of non-ferrous metals in the MF is also relatively small. The utilizability of lead (uPb) in MF is 9.14%, while uZn is 7.36%. uCu is relatively high (20.42%) due to the high Cu concentration in this fraction, but the utilizability of Fe is low (16.79%). Non-ferrous metals exhibited high utilizability in the NMF. uPb is 90.86%, even though the Pb concentration (1.78%) in proportion to the input (1.67%) is only 6.7% higher, indicating a very low concentration. Concentrations of Zn and Cu in the NMF are 7.61% and 0.42%, respectively. The NMF had a lower concentration of Cu than the input. The Fe content in the NMF is significant at 30.01%, indicating a minor decrease from the 30.72% of Fe in the input raw material. The utilizability of Zn in the NMF is 92.64%. However, the Zn content (7.61%) in the NMF in relation to input has increased by 8.7%, which is quite low. 

In comparison to the input (30.72%) and NMF (30.01%), the quantification of Fe in the MF indicates a low concentration (34.83%). Iron is found in the glassy phase, spinels, franklinite, and wüstite. In a low magnetic field, one part of these mineral forms is transferred into the MF and the other part remains in the NMF, depending on their magnetic properties. However, the iron content in the MF is only 13.4% higher than that of the input. The MF’s insufficient uFe (16.79%) indicates 83.21% loss of Fe in the NMF. Thereby, there was no discernible separation of non-ferrous metals from iron carrier components at a low magnetic field of 0.07 T. Non-ferrous metals were partially isolated from the MF (1.03% Pb, 3.48% Zn, and 0.62% Cu), and therefore lost from further refinement procedure. Furthermore, their presence deteriorates the quality of the fraction in which Fe should be concentrated. A very small concentration of Fe in MF (34.83%) emerged under a low magnetic field. Given the same magnetic field settings, the concentration of Cu (20.42%) in the MF was high, which is not ideal from the standpoint of the product’s quality. 

Pb-Zn slag separation under low magnetic field conditions was not successful, according to the data presented. More specifically, the Cu content in this fraction was lower than that of the input raw material, and no substantial concentration of non-ferrous metals in NMF was achieved relative to the input. There was no noticeable increase in iron concentration in MF in comparison to the input.

#### 3.2.2. Magnetic Separation of Pb-Zn Slag by Disk Separator 

**Figure 8:**
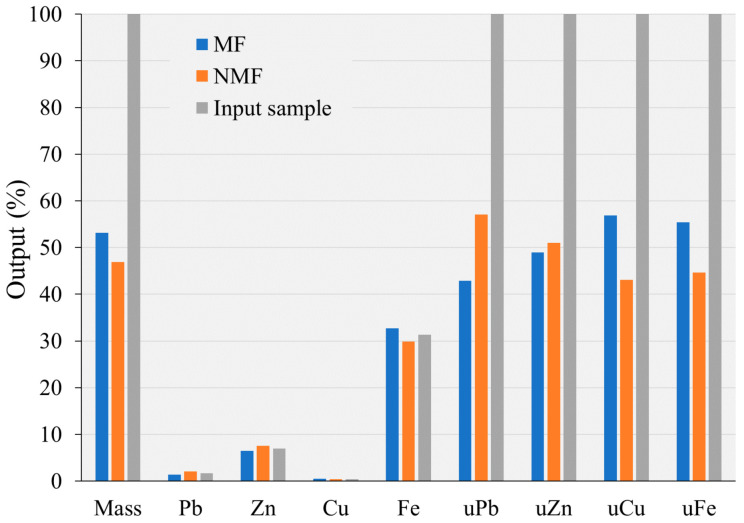
Magnetic separation balance of Pb-Zn slag after application of disk separator.

The second MS experiment was performed using a magnetic separator with a disk. Separation was conducted in a dry state, with a magnetic induction more than 15 times higher than that of a Davis separator. The MF and NMF of the Pb-Zn slag sample were obtained following the disk separation test and both fractions were chemically analyzed by the AAS. The results are presented in Figure 8 as a material balance. Recovery per element is calculated from the mass of starting sample (input, NMF and MF, i.e., the initial stacks in Figure 8).

The results displayed in Figure 8 show that the separation of the MF and NMF was more successful than in the previous experiment, but still not perfect, even with a disk separator with strong magnetic induction (1.1 T). The mass of MF acquired in this experiment was 53.11%, which is significantly higher than the mass obtained by Davis separator (14.81%). The MF comprised comparatively larger concentrations of non-ferrous metals: 1.38% Pb, 6.44% Zn, and 0.49% Cu. This separation resulted in higher Pb and Zn concentrations in the MF compared to the experiment with a Davis separator (Figure 7). Cu concentration in the input (0.46%) was lower than that of MF (0.49%). The copper concentration in the MF is undesirable in terms of the product’s quality. In contrast to the content obtained by the Davis separator, which operated in a low magnetic field (0.07 T), the amount of non-ferrous metals in the MF increased under a strong magnetic field (1.1 T). For instance, the Zn content in the MF was 6.44%, which is slightly lower than the Zn concentration in the input sample (6.98%) and far higher than the Zn content obtained by the Davis separator (3.48%). The appearance of Zn in the MF can also be partially attributed to the presence of franklinite (ZnFeO_4_) or amorphous forms that are chemically similar to franklinite. 

The mass share of MF after the experiment in the disk separator is nearly 3.6 times higher than the mass obtained via Davis separator. This suggests a notable loss or use of non-ferrous metals. The utilizability of Pb (uPb) is 42.9%, while uZn, and uCu are 48.97% and 56.92%, respectively. Utilizability percentages of the non-ferrous metals in the NMF are as follows: uPb is 57.1%, uZn is 51.03%, and uCu is 43.08%. Despite a 1.218 and 1.088-fold increase in Pb and Zn concentrations relative to the input(s), respectively, these levels remain low. The concentration of Zn in the NMF of the sample treated with the Davis separator is approximately the same as the Zn concentration obtained by the disk separator, but the utilizability of Zn in the NMF of the Davis separator is significantly higher than that of the disk separator. Similar to uZn, utilizability of Cu in NMF of Davis separator was higher than that of disk separator, even though Cu concentrations in both NMFs were the same.

Compared to the input, the concentrations of Pb (2.08%) and (7.6%) Zn in the NMF were higher. The amount of Cu (0.42%) in this fraction decreased in comparison to the input (0.46%). Furthermore, Fe content in the NMF is significant at 29.86%, exhibiting a small decrease from the Fe value (31.38%) obtained for the input raw material. The low concentration of iron in the MF (32.72%) in comparison with input and NMF can be explained by the fact that in a strong magnetic field, fewer magnetic minerals with lower iron content, such as spinels and franklinite composed of various divalent and trivalent cations (in this case, Fe and Zn), passed into the magnetic fraction. 

When franklinite with a high Zn content is submitted to separation using a strong magnetic field, the Zn content in the MF increased. Weaker magnetic minerals with low Fe concentration were also separated, influencing an increase in Fe content in the magnetic fraction. In this case, Fe is concentrated in the MF with a small increase (4.3%) over the input. The utilizability of Fe (uFe) in the MF is 55.38%, resulting in a Fe loss of 44.62% in the NMF. Thus, using a stronger magnetic field (1.1 T) in the Pb-Zn slag separation process did not result in significant separation of non-ferrous metals from iron carrier components. 

#### 3.2.3. Mineralogical Analysis of Magnetic Separation Products—Optical Microscopy

According to the results of chemical analyses, i.e., balance analysis, the magnetic separation applied to the Pb-Zn slag in two previously explained testing sets with different input conditions and magnetic induction strengths did not yield a suitable technological outcome. A mineralogical analysis of the resulting magnetic separation products was carried out in order to gain a better understanding of why this procedure, which was intended to separate non-ferrous metals from components containing iron, underperformed.

The structural elements of the analyzed samples are grouped into five formations according to the degree of freedom and the way they interconnect: (1) free grains—representing free mineral grains with about 100% visible surface; (2) inclusions—represent the examined mineral, which contains other minerals whose total surface area is not exceeding 10–30%; (3) impregnations—represent the examined mineral, which is incorporated in other minerals where its total surface is not exceeding 10–30%; (4) simple fusion—represents the examined mineral, which is fused with one mineral, where its total surface ranges from 30–70%; and (5) complex conglomerate—represents the examined mineral, which is conglomerated with several minerals and its total surface ranges from 10–50%.

**Figure 9:**
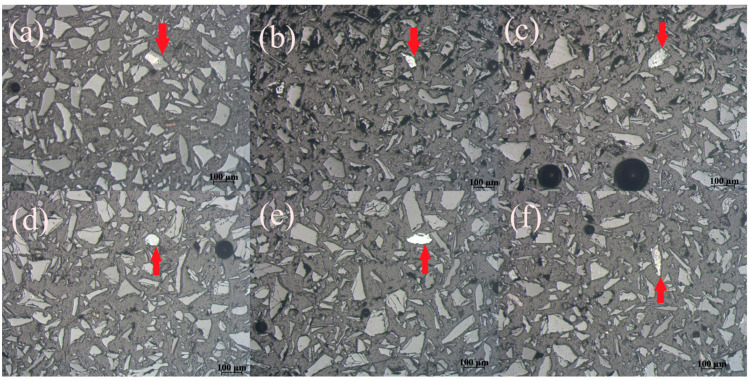
Microphotographs of the Pb-Zn slag’s MF: (**a**) simple fusion; (**b**–**e**) free alloy grain; and (**f**) simple fusion.

Microphotographs of the Pb-Zn slag’s MF sample obtained on a Carl Zeiss-Jena’s JENAPOL-U polarizing microscope are shown in Figure 9a–f. All microphotographs were recorded in air and reflected light. The estimated percentage of free alloy grains in MF is 91%, while the percentage of alloy grains that are in the form of inclusions, impregnations, or simple fusions sums up to 9%. The size of the free grains ranges from 26 µm to 159 µm, while the diameters of inclusions, impregnations, or simple fusions are from 50 µm to 74 µm. The inclusions whose dimensions range from a maximum of 20 µm to submicron dimensions are not included in the calculation because their mass share is small and it was not possible to separate them.

Figure 9a gives a preview of a simple fusion of two minerals combined in a single formation (marked with red arrow). The formation is angular, at approximately 120 µm in size. The bigger mineral formation is of grey color, while the smaller fused mineral (visible on the surface of the formation) is of shiny white color. Due to the light reflection, it can be assumed that this mineral formation might represent the conglomeration of metallic minerals. The free alloy grains are visible in Figure 9b–d (marked with red arrows). The grain in Figure 9b is small and white with a shiny surface. Its shape is angular, and its diameter is less than 100 µm. The alloy grain visible in Figure 9d is bigger, at about 130 µm in size. Its edges are more roundly shaped, and its color is off-white with lesser reflection. The metallic grain in Figure 9d is perfectly round-shaped, clearly white, with a diameter of approximately 60 µm. The isolated alloy grain in Figure 9e is elongated and white, with its horizontal dimension being around 130 µm, and its vertical dimension 20–30 µm. Simple fusion identified in Figure 9f (marked with red arrow) is needle-like. Its vertical dimension is approximately 150 µm, while its horizontal dimension is 10 µm. The formation is specked with grey and white small mineral inclusions.

**Figure 10:**
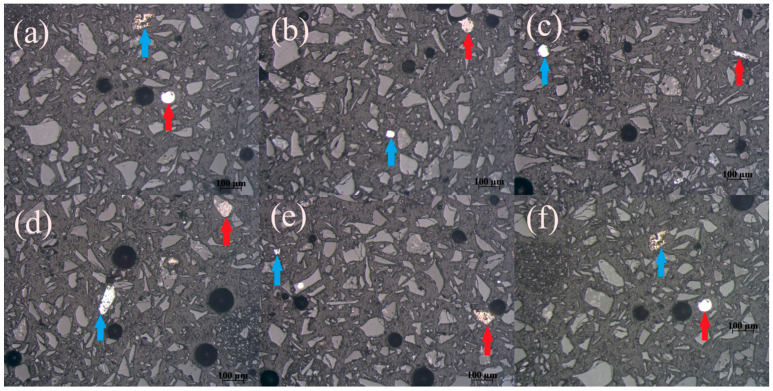
Microphotographs of the Pb-Zn slag’s NMF: (**a**–**d**) free alloy grains; (**e**,**f**) free alloy grain and simple minerals’ fusion.

Microphotographs of the Pb-Zn slag’s NMF are given in Figure 10a–f. All microphotographs were recorded in air and reflected light. The percentage of free alloy grains in this sample is 83%, while the percentage of alloy grains that are in the form of inclusions, impregnations, or simple fusions is 17%. The diameters of the free grains range from 35 µm to 140 µm. The inclusions, impregnations, or simple aggregates exhibited diameters in the range of 32 µm to 92 µm. Inclusions with dimensions from 20 µm to submicron dimensions are not included in the calculation. It was observed that the alloy content in NMF is higher compared to MF. 

Figure 10a gives a preview of two free alloy grains. The grain marked with a red arrow is round, white, and shiny. Its diameter is approximately 80 µm. The grain marked with a blue arrow is angular, of brown and white color, and approximately 100 µm in size. The alloy grains in Figure 10b are both roundly shaped. The grain marked with the blue arrow is smaller (15 µm), while the other grain is bigger (50 µm) and specked with brownish dots. Figure 10c represents two independent metallic grains, of which one is white and round with a diameter of 20 µm (blue arrow) and the other (red arrow) is elongated, its length being approximately 130 µm and of grey-white color. Two free grains represented in Figure 10d are both comparatively bigger than the rest of the alloy grains identified in Figure 10. Their diameters are over 100 µm. The grain marked with blue arrow is white, specked with brown dots, while the grain marked with red arrow is of beige to light brown color. Figure 10e gives a preview of a very small metallic grain (blue) and bigger mineral fusion composed of black, brown, and white inclusions. One small round, white metallic grain can be seen in Figure 10f (red arrow), as well as fusion of minerals (blue arrow) composed of grey and light brown mineral inclusions.

The analysis of the Pb-Zn slag’s MF and NMF microphotographs confirmed that this artificial raw material was adequately prepared for the magnetic separation process. In theory, the liberation of magnetic particles should be optimal. Specifically, the MF contained 91% free alloy grains, while only 9% of them coalesced with other grains through inclusions, impregnations, simple fusions, or complex conglomerates. Given that 91% of the non-ferrous metal alloy grains (copper, zinc, and lead) found in the MF are free, they ought to have passed into the MF during the MS procedure. The degree of freedom of the alloy grains in this sample is ideal, based on the visual assessment of the NMF microphotographs. The analysis of the test data showed that 83% of the alloy grains in the NMF are free, whereas 17% are fused in different ways with other grains. This indicates that free alloy grains of non-ferrous metals (Pb, Zn, or Cu) account for 83% of all the alloy grains found in NMF and that these grains entered the NMF during the magnetic separation process. Thereby, mineralogical analysis revealed that the Pb-Zn slag was adequately prepared for magnetic separation process. Grains derived from various slag components were optimally released because the input material was crushed to the theoretically ideal grain size class of −100 + 0.0 µm.

#### 3.2.4. Scanning Electron Microscopy Analysis of Pb-Zn Slag Samples

**Figure 11:**
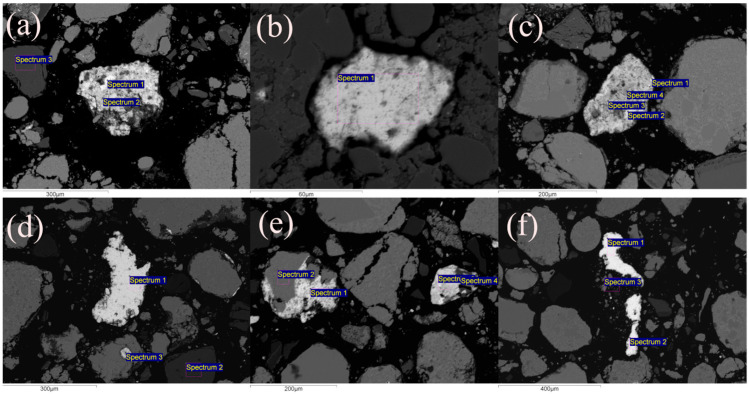
SEM microphotographs of Pb-Zn slag: (**a**) oxidized lead alloy grains; (**b**) glassy matrix; (**c**) oxidized lead alloy grain; (**d**) oxidized lead alloy grains and oxidized lead–zinc alloy grain; (**e**) oxidized copper–zinc alloy grains; and (**f**) oxidized lead alloy grains.

As it was previously concluded, the magnetic separation of the Pb-Zn slag sample using two distinct magnetic separators with different magnetic inductions produced unsatisfactory results. The chemical analysis of the outputs revealed that there was no adequate separation of non-ferrous metals into the NMF and iron-containing components into the MF. To better understand this problem and the outcomes, scanning electron microscopy, accompanied by an EDS system for targeted chemical analysis, was performed on the initial slag sample to identify the presence of certain elements in individual slag grains. The SEM microphotographs of characteristic Pb-Zn slag grains are given in Figure 11a–f. Table 1 shows the semiquantitative chemical analysis (EDS) of Pb-Zn slag grains.

As it can be seen from Table 1, the first analyzed grain of the oxidized lead alloy (Figure 11a), indicated as 11a/1 in Table 1, has 75.23% Pb, 0.79% Cu, and 0.70% Fe. Although this percentage of iron is very low, it provides a certain degree of magnetism. The analysis conducted on the second position of the same grain revealed a similar composition: 70.73% Pb, 1.77% Cu, and 3.76% Fe. The iron content was somewhat higher; thus, it contributed to the magnetism of the grain. The second analyzed grain of oxidized lead alloy (11a/3) had a lower Pb content (54.98%), a higher percentage of Cu (17.02%), and 3.74% of Fe, which contributed to the grain’s magnetism.

The grain (11b/1, Figure 11b) corresponding to the glassy matrix contained 7.03% Ca, 8.26% Si, and 29.05% Fe, as well as a large amount of copper (0.60%) and zinc (16.67%). Due to its high Fe concentration, this grain can be considered magnetic. Therefore, Cu and Zn constituents are transported with this grain and concentrated into the magnetic fraction, as seen in both magnetic separation studies. The oxidized lead alloy grain, designated as 11c/1–4, is identified in Figure 11c. The initial EDS spectrum (11c/1), in addition to the non-ferrous metals (82.69% Pb and 2.36% Cu), is iron-free and thus nonmagnetic. EDS spectra 11c/2 and 11c/3 confirmed this result, as can be seen in Table 1. The fourth spectrum (11c/4) comprised very low Fe concentration (0.95%) in addition to the non-ferrous metals (79.05% Pb and 3.50% Cu). The iron content is minimal, although the grain can be described as low-magnetic. Figure 11d represents two oxidized lead alloy grains and an oxidized lead-zinc alloy grain. The grain 11d/1 contains non-ferrous metals (84.62% Pb and 2.36% Cu) but does not contain iron in its composition. The spectrum of the other grain (11d/2) revealed a similar composition (Table 1). As such, these grains are undoubtedly non-magnetic. The third grain (11d/3) in addition to lead (76.23%) has 0.54% Fe, which makes it slightly magnetic. Figure 11e shows two oxidized copper–zinc alloy grains. Their compositions are similar, as can be seen from EDS analysis: Cu accounted for approximately 32–38% and Zn for 30–35%, and the content of Fe is significant at approximately 8–10%, while the percentages of Ca, Si, and Al are below 3%. These grains can be defined as magnetic. The presence of Ca, Al, and Si indicates that grain is probably fused with a certain amount of glassy phase. The last two investigated grains (11f/1–3, Figure 11f) are non-magnetic because they are predominantly composed of non-ferrous metals (87–91% Pb and 0.3–0.45% Cu). These grains contain certain amounts of silver (0.97–1.22% Ag).

The EDS analysis of the alloy grains and the glassy matrix indicate that non-ferrous metal grains have entered the magnetic phase. To be more specific, the grains of non-ferrous metal alloys containing iron show a certain degree of magnetism, and as such, they pass into the magnetic fraction during magnetic separation. These results demonstrated why the magnetic concentration of Pb-Zn slag did not show the expected results. The majority of Fe was bound in mineral or amorphous formations; therefore, it was impossible to liberate it via a mechanical procedure for concentration. As a result, the usable components could not be separated from the tailings to form an individual product. 

### 3.3. Gravity Concentration of Pb-Zn Slag

The investigations were then directed toward separating and valorizing useful elements from Pb-Zn slag using the gravity concentration method. Several factors were explored before determining the most suitable GC settings. The key premise is that slag is not an ore, which implies that it contains more than just minerals. The methodology of the experiment was determined by the sample’s mineralogical properties (the presence of amorphous and crystalline phases), structural and textural characteristics, degree of cohesion (freedom) of components, and the fact that the sample contains useful components primarily as alloys. Namely, Pb-Zn slag consists of alloy–mineral intergrowths and an amorphous phase [1]. The investigated slag is composed of: (1) oxidized lead alloy with copper and iron (Pb, Cu, O, Fe): γ = 9.84–9.25 g/cm^3^; (2) oxidized lead alloy with copper (Pb, Cu, O): γ = 8.73–9.21 g/cm^3^; (3) oxidized zinc alloy with copper (Cu, Fe, Zn, O): γ = 6.33–6.73 g/cm^3^; (4) wüstite grains: γ = 5.7 g/cm^3^; (5) elemental iron grains: γ = 7.87 g/cm^3^; (6) galena grains (PbS): γ = 7.2–7.6 g/cm^3^; (7) sphalerite grains (ZnS): γ = 3.9–4.1 g/cm^3^; (8) grains of amorphous phase (spinel) chemically similar to franklinite: γ = 5.07–5.22 g/cm^3^; and (9) silicate grains (gelenite/akermanite/wollastonite): γ = 3.00 g/cm^3^. The initial Pb-Zn slag sample had the same characteristics as the input sample for magnetic separation, with a particle size of −100 + 0.0 µm. These tests marked the first attempts to conduct gravity concentration and the subsequent valorization of Pb-Zn slag “Veles”. Because there are no predetermined standards, requirements, or attributes that must be met by the product made by concentrating Pb-Zn slag in order for it to be commercially viable, the success of gravity concentration cannot be evaluated solely on the basis of the tests conducted.

#### 3.3.1. Concentration Criterion for Gravity Concentration Experiment

If the concentration criterion is greater than 1.5 (*ζ* > 1.5), the probability of successful gravity concentration of a raw material in a wet environment (water), i.e., mineral/component separation into different products, is high. The GC procedure was limited to obtaining two products, ∆T and ∆L, because the Pb-Zn slag had a metal alloy concentration of roughly 5–6% (i.e., tailings content is slightly more than 90%). As an output, the total raw material was separated into two parts: ∆L (the tailings fraction) and ∆T (the concentration of non-ferrous metal alloys). 

The following formula is used to calculate the separation of a two-component system, including grains of oxidized lead–copper alloy and an amorphous phase (spinel), when the concentration conditions for their GC in a thin layer of water are satisfied:

(1) $$\zeta_{1}=\frac{\gamma_{alPb-Cu}-\gamma_{H_{2}O}}{\gamma_{amorph.\;}-\gamma_{H_{2}O}}=\frac{7.9}{4.1}=1.93$$

Separation in a two-component system consisting of oxidized zinc–copper alloy grains and an amorphous phase is conducted using the formula:

(2) $$\zeta_{2}=\frac{\gamma_{alZn-Cu}-\gamma_{H_{2}O}}{\gamma_{amorph.\;}-\gamma_{H_{2}O}}=\frac{5.6}{4.1}=1.40$$

The experiment used a concentration criterion of 1.5 due to the higher first concentration criterion (*ζ*_1_ = 1.93) and lower second concentration criterion (*ζ*_2_ = 1.4) compared to the theoretical value required for the successful separation of two minerals or components.

The separation of the copper-containing amorphous phase (spinel) grains from the oxidized zinc alloy grains is challenging. The worst-case scenario is adopting the amorphous phase’s specific mass as γ = 5.07–5.22 g/cm^3^, which is also the specific mass of franklinite and magnetite. The amorphous phase might be similar to franklinite, but in reality, this component of slag is far more complex, containing Si, Ca, Mg, and Al. The amorphous phase has a specific mass of less than γ < 5.0 g/cm^3^; however, a larger value (5.6 g/cm^3^) was chosen for safety.

#### 3.3.2. Grain Size Distribution of the Pb-Zn Slag Used for Gravity Concentration

**Figure 12:**
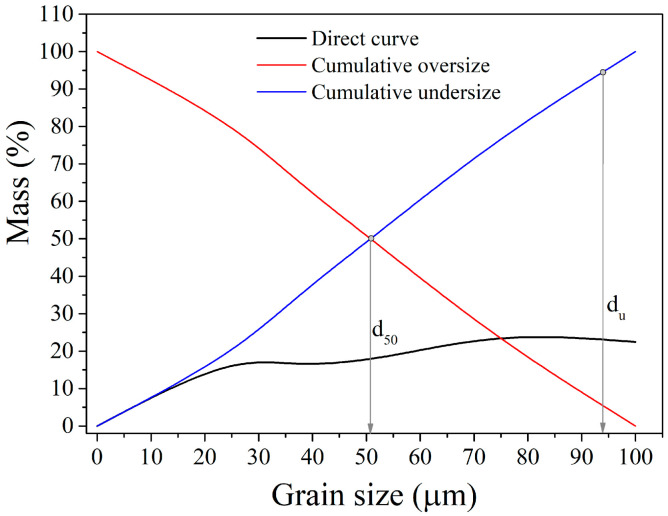
Grain size distribution of the PB-Zn slag sample used for gravity concentration.

Five grain size classes obtained by the wet sieving were employed in the GC procedure: −100 + 75 µm; −75 + 53 µm; −53 + 37 µm; −37 + 25 µm; −25 + 0.0 µm. The grain size distribution of the sample is presented in Figure 12.

The initial Pb-Zn slag sample for GC has a mean grain diameter of d_50_ = 50.86 μm and a maximum diameter of d_95_ = 94.82 μm. Even after the sample was ground to a size of −100 + 0.0 µm, 18.88% of the finest class (−25 + 0.0 µm) was present. A high percentage of the valuable components were softer than the tailings and were easily crushed, ending in the finest classes, which is undesirable. The classes −100 + 75 µm, −75 + 53 µm, −53 + 37 µm, and −37 + 25 µm contributed 22.45%, 25.11%, 17.79%, and 15.77% of the overall mass share, respectively.

#### 3.3.3. Load Balance of the Gravity Concentration of Pb-Zn Slag

**Figure 13:**
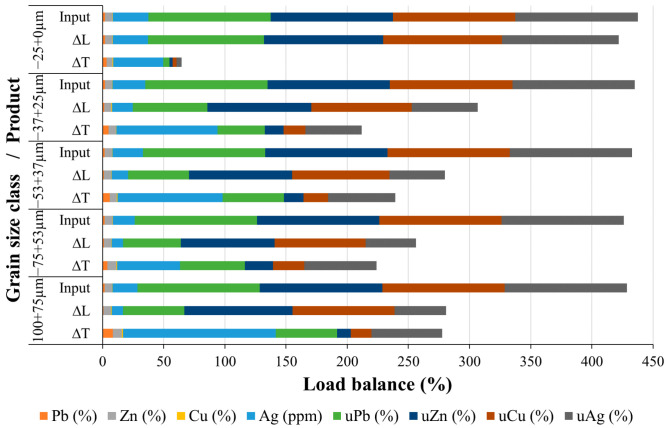
Load balance of the GC of Pb-Zn slag in relation to grain size class.

**Figure 14:**
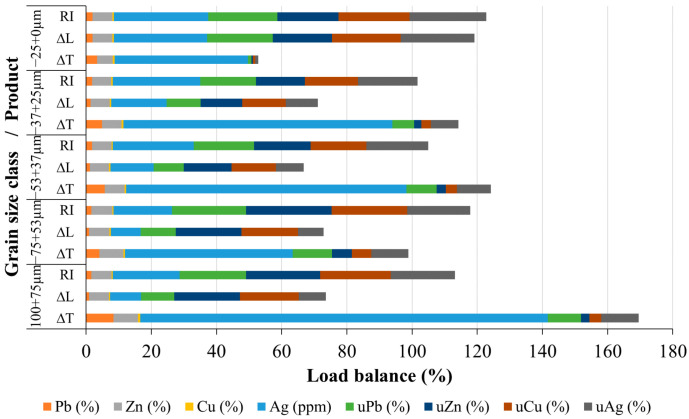
Load balance of the GC of Pb-Zn slag in relation to the input.

Gravity concentration was conducted on a Wilfley 13 shaking table (described in Section 2.6). When using the GC approach, it is difficult to visually distinguish the fractions of the slag by color (there is a visible separation on the table, i.e., bands are distinguishable, but the color difference is minimal), which is very different from ores. Sub-samples were taken from each grain-size class and processed via shaking table. Upon GC, all acquired compounds were dried, their masses determined, and samples prepared for chemical analysis. Based on the chemical analysis, a load balance was determined, demonstrating the concentrations and utilizability values of all the obtained products. Figure 13 and Figure 14 illustrate the load balance based on grain size classes and input, respectively. Figure 13 displays the load balance for each class, which equals 100%. Figure 14 shows the load balance, which takes into consideration all classes and products in proportion to their input. In addition, Table 2 shows the cumulative products of GC given as metal (Pb, Zn, Cu, and Ag) concentrations and their utilizabilities.

Based on the data illustrated in Figure 13 and Figure 14, it can be concluded that the separation of individual components into different products based on the grain size classes using the concentration criterion is well performed. In the coarsest class (−100 + 75 µm), 9.46% of the total mass belongs to the ΔT, i.e., metal concentrate fraction. The remaining 90.45% of the mass is in the tailings fraction (ΔL). Alloy grains are easily separable in this class. ΔT fractions are 20.44%, 15.79%, and 14.97% for the three following grain size classes (−75 + 53 µm, −53 + 37 µm, and −37 + 25 µm). Useful components summed up to only 3.03% in the finest class (−25 + 0.0 µm), which means that this class can easily be disregarded as mainly tailings. This demonstrates that the gravity concentration parameters used in this experiment were correctly determined and implemented.

Figure 13 and Figure 14 show satisfactory results for separating lead and silver concentrates (ΔT fractions of Pb and Ag). The results obtained for the separation of zinc and copper concentrates (ΔT fractions of Zn and Cu) are somewhat inferior. As it can be seen from Figure 13, the coarsest class (−100 + 75 μm) produced the highest concentrations of Pb, Zn, Cu, and Ag. Their ΔT fractions are: 8.44%, 7.42%, 0.77%, and 125 ppm (g/t), respectively. Smaller grain size classes gave lower concentrates (ΔT) of non-ferrous metals and silver. For instance, in the finest class (−25 + 0.0 µm) the following concentrations of Pb, Zn, Cu, and Ag were identified: 3.37%, 4.82%, 0.54%, and 40.88 ppm. The silver quantity reached a minimum of 51.5 g/t in the −75 + 53 μm class. Concentrations of Zn ranged from 5% to 8% in useful grain size classes, while Cu was below 1% in all classes. Thereby, the majority of useful elements are located in the coarser classes, from where they can be easily separated.

The content of lead in the cumulative concentrate (i.e., all ΔT fractions combined) is 5.28% (Table 2), with the Pb utilizability being 39.22%. The content of silver in the cumulative concentrate is 76.12 g/t, with the Ag utilizability being 42.68%. The results regarding the content and utilizability of lead and silver obtained during gravity concentration indicate that silver follows lead, that is, there is free silver and invisible silver in the structure of the lead alloy intergrowths [37]. Free silver, which is very fine, at 2–5 μm in size, probably passed into the finest class (−25 + 0.0 μm) during comminution and ended up in tailings. As it was mentioned, the gravity concentration procedure was more successful in the separation of Pb and Ag than Cu and Zn. For each grain size class, the Zn concentrates (ΔT) were higher than its tailings fraction (ΔL), except for the finest size class (−25 + 0.0 μm), in which ΔL was higher than ΔT. The Cu content for each grain size class exhibited a higher ΔT fraction than ΔL fraction. This means that separation has been successfully achieved. The Zn content in the cumulative concentrate (all ΔT fractions combined) is 6.69%, with a utilizability of 14.16%. The Cu content in the cumulative concentrate is 0.58%, with an utilizability of 16.58%. The GC underperformance with zinc and copper can be attributed to the small concentration criteria of 1.4 in the two-component system of oxidized zinc alloy grains with copper and an amorphous phase.

## 4. Conclusions

This study proposed a new processing route for valorizing historical Pb-Zn slag. The procedure involves the mechanical means of separation and concentration of valuable non-ferrous metals (Pb, Zn, Cu, and Ag): crushing, grinding, magnetic separation, and gravity concentration. This technology could have a considerable impact on the global beneficiation of metallurgical slags since, in theory, it is much simpler, more ecologically friendly, and less expensive than typical pyrometallurgical or hydrometallurgical methods. The main conclusions are summarized below:

− This is the first all-inclusive (physical–chemical, mineralogical, and technical) investigation into this type of Pb-Zn slag. There are no past practical or theoretical experiences with this specific raw material; therefore, the findings cannot be compared to those achieved with ores, minerals, or even other forms of slag. For these reasons, the techniques for preparing and valorizing beneficial components are required to be tailored to the specific tested raw material, which represents a unique study in this field of science.
− The studied Pb-Zn slag is not an ore but rather an engineered secondary raw material; therefore, it comprises more than just minerals. The identified minerals are largely artificial minerals, rare in nature, and generated under extreme conditions. The slag consists of an amorphous phase and alloy grains of lead, copper, and zinc, which are valuable components. Minor silver inclusions are also present.
− Grindability tests (14-to-44 min sequences, wet grinding) demonstrated that coarse grain size classes (+150 μm, −150 + 100 μm) could not be discarded as tailings since they include valuable alloy grains or alloy–glassy phase intergrowths. During grinding, there was no significant segregation of non-ferrous metals into finer grades from which concentration could not be accomplished. The mineralogical examination of the grindability test results revealed that the Pb-Zn slag’s usable components are the most efficiently liberated at a crushing fineness of −100 + 0.0 µm.
− The magnetic separation performed on the Davis and disk separators gave unsatisfactory results because the magnetic and non-magnetic fractions could not be adequately separated. Davis separators produced MF in the amount of 14.81%, while the NMF accounted for 85.19%. The NMF showed no significant concentrations of non-ferrous metals, but the MF had a rather large concentration. Disk separator gave better results regarding MF (53.11%), but the Fe level in NMF was high (29.86%). The optimal liberation of non-ferrous elements via MS was prevented because alloy grains are, to a large extent, merged with aluminosilicate matrix and iron-bearing minerals, forming pseudo-magnetic intergrowths and passing into the magnetic fraction. As a result, the usable components could not be separated from the tailings to produce an adequate end product.
− Gravity concentration gave satisfactory results regarding Pb and Ag liberation. The majority of useful elements are located in the coarser classes, from where they can be separated. The GC performed poorly with Zn and Cu due to intergrowths of oxidized zinc alloy grains with copper and an amorphous phase from which mentioned elements could not be concentrated.
− Both magnetic separation and gravity concentration have limitations: MS performed better in Zn liberation, whilst GC was better at concentrating Pb and Ag. Instead of applying these methods as a single-stage refining processes, it may be more efficient to use them as subsequent treatments in a more advanced concentration procedure.

This is the first study to investigate the feasibility of this novel approach of valorizing Pb-Zn slag, which includes the direct testing of the slag using various mechanical separation and concentration techniques. This technology will be able to replace and thereby reduce the use of hazardous chemicals employed in hydrometallurgical refinement processes and the extreme energy consumption and CO_2_ emission characteristics of pyrometallurgical treatments. After the valuable metals have been extracted, the amorphous aluminosilicate fraction of the Pb-Zn slag can be reused as an alternative raw material in the construction industry, enhancing the circularity of the proposed method.

## Figures and Tables

**Table 1:** Semi-quantitative EDS analysis of the selected grains in Pb-Zn slag sample.

Spectrum in Figure 11	Pb (%)	Cu (%)	Fe (%)	As (%)	S (%)	O (%)	Ca (%)	Si (%)	Al (%)	Zn (%)	Ag (%)
11a/1	75.23	0.79	0.70	-	-	22.40	-	-	-	-	-
11a/2	70.73	1.77	3.76	1.1	13.30	8.79	-	-	-	-	-
11a/3	54.98	17.02	3.74	1.1	13.00	8.15	-	-	-	-	-
11b/1	-	0.60	29.05	-	-	30.30	7.03	8.26	2.45	16.67	-
11c/1	82.69	2.36	-	-	-	14.81	-	-	-	-	-
11c/2	83.25	1.42	-	-	-	13.78	-	-	-	-	-
11c/3	81.85	2.03	-	-	-	15.25	-	-	-	-	-
11c/4	79.05	3.50	0.95	-	-	16.35	-	-	-	-	-
11d/1	84.62	2.36	-	-	-	13.01	-	-	-	-	-
11d/2	68.78	0.87	-	-	-	14.52	-	-	-	15.13	-
11d/3	76.23	-	0.54	-	-	21.81	1.41	-	-	-	-
11e/1	-	36.97	8.94	-	-	12.81	2.53	3.56	1.28	33.1	-
11e/2	-	35.84	9.11	-	-	15.57	1.95	2.97	1.29	30.9	-
11e/3	-	37.12	8.85	-	-	14.65	2.04	3.75	1.32	31.14	-
11e/4	-	32.55	9.14	-	-	16.12	2.13	3.14	1.15	35.54	-
11f/1	90.70	0.46	-	-	-	7.76	-	-	-	-	0.97
11f/2	88.56	0.32	-	-	-	7.98	-	-	-	-	1.12
11f/3	87.95	0.41	-	-	-	8.58	-	-	-	-	1.22

**Table 2:** Cumulative balance of gravity concentration products.

Grain Size Class (μm)	Product	M (%)	Pb (%)	Zn (%)	Cu (%)	Ag (ppm)	uPb (%)	uZn (%)	uCu (%)	uAg (%)
−100 + 0	∑∆T	12.99	5.28	6.69	0.58	76.12	39.22	14.16	16.58	42.68
	∑∆L	87.01	1.22	6.05	0.43	15.26	60.78	85.84	83.42	57.32
	Input	100.00	1.75	6.13	0.45	23.17	100.00	100.00	100.00	100.00

## Data Availability

The original contributions presented in the study are included in the article, further inquiries can be directed to the corresponding author.
